# Composition, Diversity, and Stability of Microbial Assemblages in Seasonal Lake Ice, Miquelon Lake, Central Alberta

**DOI:** 10.3390/biology2020514

**Published:** 2013-03-27

**Authors:** Anna Bramucci, Sukkyun Han, Justin Beckers, Christian Haas, Brian Lanoil

**Affiliations:** 1 Department of Biological Sciences, University of Alberta, Edmonton, AB T6G2E9 Canada; E-Mails: bramucci@ualberta.ca (A.B.); bada.han@gmail.com (S.H.); 2 Department of Earth and Atmospheric Sciences, University of Alberta, Edmonton, AB T6G2E9 Canada; E-Mails: beckers@ualberta.ca (J.B.); haasc@yorku.ca (C.H.)

**Keywords:** seasonal lake ice, Miquelon Lake, bacterial diversity, eukaryotic diversity, seasonal dynamics, winter-over dynamics

## Abstract

The most familiar icy environments, seasonal lake and stream ice, have received little microbiological study. Bacteria and Eukarya dominated the microbial assemblage within the seasonal ice of Miquelon Lake, a shallow saline lake in Alberta, Canada. The bacterial assemblages were moderately diverse and did not vary with either ice depth or time. The closest relatives of the bacterial sequences from the ice included Actinobacteria, Bacteroidetes, Proteobacteria, Verrucomicrobia, and Cyanobacteria. The eukaryotic assemblages were less conserved and had very low diversity. Green algae relatives dominated the eukaryotic gene sequences; however, a copepod and cercozoan were also identified, possibly indicating the presence of complete microbial loop. The persistence of a chlorophyll *a* peak at 25–30 cm below the ice surface, despite ice migration and brine flushing, indicated possible biological activity within the ice. This is the first study of the composition, diversity, and stability of seasonal lake ice.

## 1. Introduction

Remote, polar floating ice systems, such as sea ice [1] and perennial lake ice [2,3,4], harbor dynamic and diverse microbial ecosystems that play important roles in the biogeochemistry, biology, and functioning of the underlying waters and surrounding environments. However, the most familiar icy environments, including the ice that forms on lakes and streams each winter in many temperate environments, have not been studied microbiologically. While there are accounts of the phytoplankton and zooplankton winter dynamics in some northern lakes [5,6], there are none describing bacterial dynamics.

Sea ice harbors algal communities that have high rates of primary productivity, with global totals estimated to be as high as 63 to 70 Tg C year^−1^ [7]. Bacterial production in sea ice is coupled to microalgae growth [8]. Bacteria might provide algae with inorganic nutrients for prolonged sympagic survival [9]. Furthermore, diverse populations of microheterotrophs (e.g., protozoans, dinoflagellates, ciliates, and amoebae) are present and active in sea ice; thus, these systems include a complete microbial loop [10,11].

Saline lakes are found on every continent on earth, with total volumes roughly equaling the volume of terrestrial freshwater lakes [12]. The hundreds of brackish to saline lakes of the Canadian Great Plains are economically, agriculturally, and ecologically important for the region [13,14]. The lakes support numerous algal species that have been extensively documented [15,16,17,18], as well as complete microbial food webs [19,20,21]. However, little is known about how the lake water organisms are influenced by the annual freeze-thaw cycles of the upper waters or the progression from being an open-water to becoming an ice-covered lake.

Here we report the first characterization of microbial diversity in seasonal lake ice. We explored the inter- and intra-seasonal shifts in microbial assemblage composition both within the lake ice and underlying lake water of Miquelon Lake, Alberta CA. This study indicates the likely degree of microbial activity occurring throughout the winter across the frozen Albertan plains, a value which has likely been sorely underappreciated. The central hypotheses of this study are as follows: (1) the seasonal lake ice has communities similar in composition to other floating ice systems (e.g., polar sea ice or perennial lake ice); (2) the distribution of some microbial populations is limited to specific depths in the ice and/or points in the season; (3) seasonally frozen lakes maintain an actively functioning ecosystem and microbial food web throughout the winter.

## 2. Methods

### 2.1. Study Site

Miquelon Lake, located in Miquelon Lake Provincial Park Edmonton, Alberta at 53.25° N, 112.90° W [14] is small (surface area: 8.72 km^2^), shallow (mean depth: 2.7 m), secluded (residence time of water: >100 years), and brackish (6–9 ppt) [14]. Miquelon Lake waters are dominated by microbial life; higher trophic levels are absent. However, algae and cyanobacteria are abundant in this mesotrophic system [14]. The lake is fully mixed until the freeze-in (Jan-April), which leads to weak stratification in the underlying waters during this time.

### 2.2. Sample Collection and Processing

Ice cores and underlying lake water samples were collected every two weeks throughout the 4-month 2009/2010 winter season. Two 9-cm-diameter ice cores were collected with a Kovacs Mark II corer (Kovacs Enterprises Inc.; Lebanon, NH): one for biological sampling and one for bulk salinity measurements. Ice thickness measurements were taken at the time of sampling by measuring the length of the ice core. Surface water samples were collected in 1 L sterile acid washed Nalgene**^®^** bottles (VWR). Two Ice Mass Balance Buoys (IMB) (MetOcean/CRREL, Darmouth, Nova Scotia, and SAMS IMB, Oban, Scotland) obtained *in situ* measurements of air and ice temperatures throughout the winter. Temperature sensors are accurate to 0.1 °C [22].

The bulk salinity core was sectioned on site into 3–4 cm pieces and placed into sterile plastic tubs to melt. Measurements of water temperature (*in situ*), lake water salinity (*in situ*), and bulk ice core melt salinity were acquired using a MultiLine^®^ IDS WTW Cond 330i conductivity meter (Wissenschaftlich-Technische Werkstätten (WTW) Inc./Xylem Inc., Weilheim, Germany), which was calibrated prior to use according to manufacturer’s specifications.

The biology core was kept frozen at −20 °C in the dark until processing. This core was aseptically sectioned into ~5 cm sections (varying from 3–8 cm, depending on natural fractures in the ice) using a flame sterilized 15 cm drywall saw. To ensure aseptic sampling procedures one test core was sectioned and melted in sheaths and four aliquots were taken from the outer ice, middle outer, middle inner, and inner most ice respectively. These sheaths were tested on DGGE and were found to be identical based on DGGE analysis, indicating no contamination; thus, no further decontamination efforts were performed. The sections were melted in the dark at 4 °C. Subsamples for cell enumeration (25 mL) and chlorophyll *a* concentrations (60 mL) were removed and prepared as described below. The remaining water (100 to 300 mL) was filtered through 0.22 μm pore size, 47 mm diameter polysulfone filters (Pall Corporation; East Hills, NY, USA). Filters were stored frozen at −80 °C in sterile sealed Seal-a-Meal**^®^** bags (Sunbeam^®^ Products Inc.; Neosho, MO, USA).

Surface water samples were kept in the dark at 4 °C and processed within 24 hours of sampling following the same procedure as the melted ice core segments. Approximately 900 mL of water was filtered for subsequent DNA processing. Brine salinity and brine volume were calculated from measured ice temperature and salinity as described previously [23].

### 2.3. Cell Enumeration

Formalin-fixed (3.7% v/v) subsamples of the ice cores and lake water were filtered on polycarbonate black membrane filters (pore size: 0.22 µm; diameter: 25 mm; Whatman; VWR) and stained with 4',6-diamidino-2-phenylindole (DAPI) (Sigma-Aldrich) for 15 minutes in the dark, and bacterial abundances were determined by fluorescence microscopy as previously described [24]. The analyses were limited to non-filamentous and non-autofluorescing bacterial morphotypes (<5 μm cell length). The volume of water examined varied from 1 to 5 mL of water, depending on the cell concentration. A procedural blank of 5 mL of sterile Nanopure water was examined to ensure sterile technique. The cell counts were run in triplicate and standard deviations ranged from 0.2 to 1 × 10^6^ cells/mL.

### 2.4. Chlorophyll a Measurements

To measure Chlorophyll *a* (Chl-*a*) concentrations, 60 mL sample aliquots were filtered though a 25 mm precombusted (500 °C for 12 h) Whatman GF/F glass fiber filter in the dark. Filters were stored frozen at −20 °C in the dark until processing. Duplicate samples were taken randomly and used as quality controls throughout the extraction and measurement process: the quality controls totaled 10% of the total number of samples. The precision of the extraction method was assessed using percent relative standard deviation (%RSD) of the duplicates, the %RSD was always below 5% for all quality control samples.

Chl-*a* was extracted by overnight incubation in 95% ethanol in the dark using a standard spectrofluorometric approach [25]. The minimum detection limit of this protocol was ~3.3 μg/L. Concentrations were determined based on a daily standard curve of Chl-*a* from *Anacystis nidulans* (Sigma).

### 2.5. Nucleic Acid Extraction

DNA was extracted using the FastDNA^®^ extraction kit according to the manufacturer’s protocol (MP Biomedicals, Solon, OH, USA). DNA was eluted in 200 µL of warm DNAse-free commercial water (Life Technologies, Grand Island, NY, USA). The solution was buffered to 1× TE concentration (10 mM Tris, pH 8.0, 1 mM NaEDTA) and stored at −20 °C.

### 2.6. Denaturing Gradient Gel Electrophoresis (DGGE)

Partial bacterial 16S rRNA genes were amplified as previously described [26] with primers 341F and 518R, with a 40-mer GC clamp on the 341F primer (GC-341f; Table 1; [27]). Eukaryotic-specific primers GC-Euk1a and Euk516r [28] were used for amplification of 18S rRNA gene (Table 1, [29,30], and references therein). All PCRs were preformed in triplicate and pooled [31].

**Table 1:** PCR primers used.

	Primer set	Target	Sequence (5'-3')	Reference
General Primers	341F	Bact 16S rRNA	CCTACGGGAGGCAGCAG	[32]
	518R	Bact 16S rRNA	ATTACCGCGGCTGCTGG	[32]
	Euk1A	Euk 18S rRNA	CTGGTTGATCCTGCCAG	[28]
	Euk516R	Euk 18S rRNA	ACCAGACTTGCCCTCC	[28]
	A21F external	Arc 16S rRNA	TTCCGGTTGATCCYGCCGGA	[33]
	344F internal	Arc 16S rRNA	ACGGGGCGCAGCAGGCGCGA	[30]
	519R internal	Arc 16S rRNA	GGTDTTACCGCGGCKGCTG	[20]
DGGE Primers	341F *	Bact 16S rRNA	CCTACGGGAGGCAGCAG	[32]
	518R	Bact 16S rRNA	ATTACCGCGGCTGCTGG	[32]
	Euk1A	Euk 18S rRNA	CTGGTTGATCCTGCCAG	[28]
	Euk516R *	Euk 18S rRNA	ACCAGACTTGCCCTCC	[28]

* GC clamp (40 bp) added for DGGE-PCR [27]. 5'-CGCCCGCCGCGCCCCGCGCCCGTCCCGCCGCCCCCGCCCC-3'.

DGGE was performed using a D-CODE system (BioRad, Hercules, CA, USA) as previously described [26]. For each sample, 400 ng of DNA were loaded. Bands were visualized after staining the gel for 15–30 minutes in SYBR Green stain (Molecular Probes, Eugene, OR, USA), according to the manufacturer’s instructions.

DGGE banding patterns were analyzed with the program GelCompar II (version 4.0; Applied Maths, Austin, TX, USA) using a 2% band position tolerance to determine band locations. The cladograms were generated using an Unweighted Pair Group Method (UPGMA) based on Dice correlation coefficients, which are based on the presence/absence of a band regardless of absolute band intensity, as previously described [26].

### 2.7. qPCR Analysis

To assess variation in relative abundance of domain-level gene copy number with time, DNA from lake ice and water samples was homogenized, resulting in one bulk sample for ice (all ice core depths and sampling dates) and one bulk sample for the underlying lake water (all sampling dates). The relative abundance of Bacteria, Eukarya, and Archaea small subunit (SSU) rRNA genes in the lake ice and lake water samples was determined using general bacterial, eukaryal, and two sets of archaeal primers (Table 1).

qPCR was performed in triplicate 10 µL reactions containing 5 µL Rotor-Gene SYBR green PCR kit (Qiagen, Inc.), 1 µM concentration of primers, 2 µL template and 1 µL Qiagen RNase-Free water. Reactions were performed in a Rotor-Gene Q (Qiagen, CA, USA) qPCR machine. PCR conditions were 40 cycles at 95 °C for 10 s and 60 °C for 15 s. Gene copy number was calculated relative to an *E. coli* genomic DNA standard for Bacterial DNA and 16S rRNA environmental gene clones for Archaea and Eukarya. Two experimental replicates were performed and data combined for analysis. Primers were tested for cross-reactivity to the standards—no cross reactivity was observed.

### 2.8. Clone Library Construction

Two lake-ice and two lake-water clone libraries were constructed: one bacterial and one for eukaryal for each. SSU rRNA genes were PCR amplified using the general primers (without GC clamps) for Bacteria and Eukarya (Table 1) as previously described [26]. PCR products were cloned using the TOPO^®^ TA Cloning^®^ Kit (Invitrogen) according to the manufacturer’s instructions. Libraries of clones were randomly selected from the Bacteria, lake ice (n = 123), Bacteria, lake water (n = 191), Eukarya, lake ice (n = 123), and Eukarya, lake water (n = 39) samples.

### 2.9. Restriction Fragment Length Polymorphism (RFLP)

Preliminary grouping of clones was performed by RFLP analysis using *HhaI* and *MspI*, as previously described [34]. Clone insert orientation was determined by unidirectional PCR with only the M13F primer in the master mix. The 5' end sequence of one representative clone for each 10 members of an operational taxonomic unit (OTU) was determined with M13F or M13R. All clones chosen for sequencing were reanalyzed via DGGE prior to sequencing to confirm band position in reference to the original samples [26]. Good’s coverage [35] was determined manually.

### 2.10. Phylogenetic Analysis

Sequences were trimmed, sections of ambiguous base pair matching were removed, and gaps were eliminated using standard methods [36]. Chimeric sequences as determined by DECIPHER [37] were excluded from analysis. Sequences were aligned in Genious 5.5.8 (Biomatters Ltd.; New Zealand) using 25 alignment iterations and the FastAligner function. All alignments were refined manually and shared gaps were eliminated. Maximum likelihood-based phylogenetic analysis was conducted with the PHYML module in Genious [38] using sequences with length ranging from 300–600 bp for the final analysis. Bootstrap support (100 iterations) is shown at the nodes.

### 2.11. Nucleotide Sequence Accession Numbers

Sequences are deposited in Genbank with the accession numbers KC592375-KC592385.

## 3. Results

**Figure 1:**
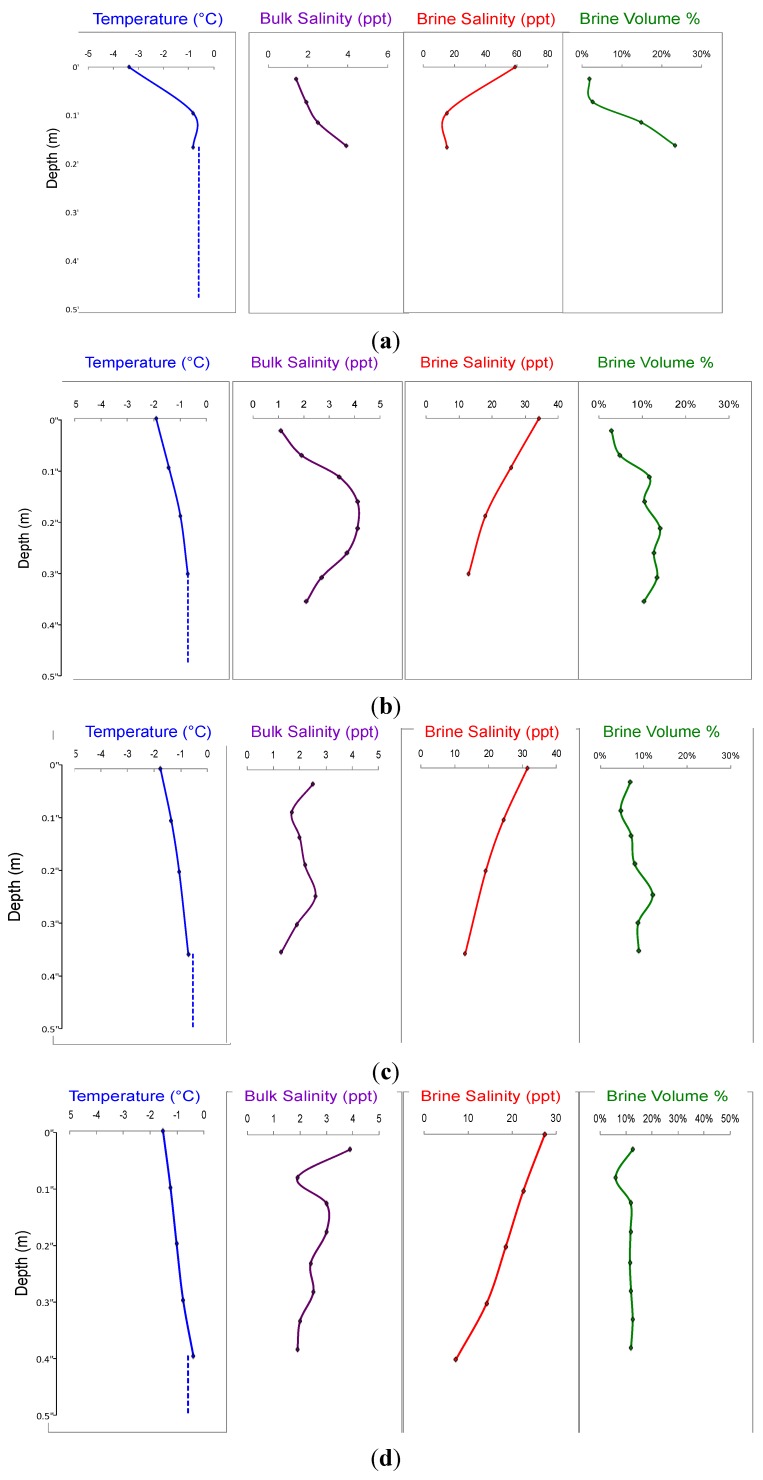
Environmental variables for representative dates during the 2009–2010 winter season at Miquelon Lake, Alberta, Canada. Solid lines are for measured parameters in ice; dashed lines are for measured parameters in the underlying water. Ice temperature and bulk salinity were measured directly. Brine salinity and brine volume were calculated from measured ice temperature and bulk ice salinity according to Cox and Weeks [23]. (**a**) 3 December 2009, (**b**) 29 December 2009, (**c**) 14 January 2010, (**d**) 11 February 2010.

Throughout the 2009–2010 winter season (November-April), air temperatures at Miquelon Lake ranged from a low of −40 °C (9 December 2013) to a high of +10 °C (29 March 2010), with an average winter air temperature of −10.6 °C. During that time period, Miquelon lake ice grew from 0 to 0.4 m in total thickness, had internal ice temperatures ranging from approximately −1 to −4 °C (Figure 1). Seasonal average ice temperatures and underlying water temperatures were very stable. Miquelon Lake water salinity ranged from 10.2 to 12.5 ppt. Brine salinity, which is directly determined by ice temperature, varied with depth, ranging from a high of 60 ppt (hypersaline, at ~1.7× higher than that of standard seawater) to a low of ~10 ppt (brackish, at ~3.5× lower that of standard seawater) (Figure 1). The brine volumes average 10% of the total ice volume throughout this season, with the lowest brine volume occurring at the same depth as the highest brine salinities (Figure 1).

Microscopic enumeration showed 3.7 × 10^6^ (±0.30 × 10^6^) cells mL^−1^ in the ice and 8.8 × 10^6^ (±10.4 × 10^6^) cells mL^−1^ in the lake water, ca. 30% of which are auto-fluorescing (and therefore likely photosynthetic) cells. With the exception of the water on 14 January 2010, which was 5× higher than other dates, the variance was <10% for all dates and depths; thus the overall average values are given. qPCR showed SSU rRNA relative gene copy number of 50% Bacteria, 50% Eukarya, and <1% for Archaea (data not shown). Note that due to differences in genome size and SSU rRNA gene copy number, this result does not indicate equal abundance or biomass of Bacteria or Eukarya; only that both Bacteria and Eukarya are abundant while Archaea are exceedingly rare.

**Figure 2:**
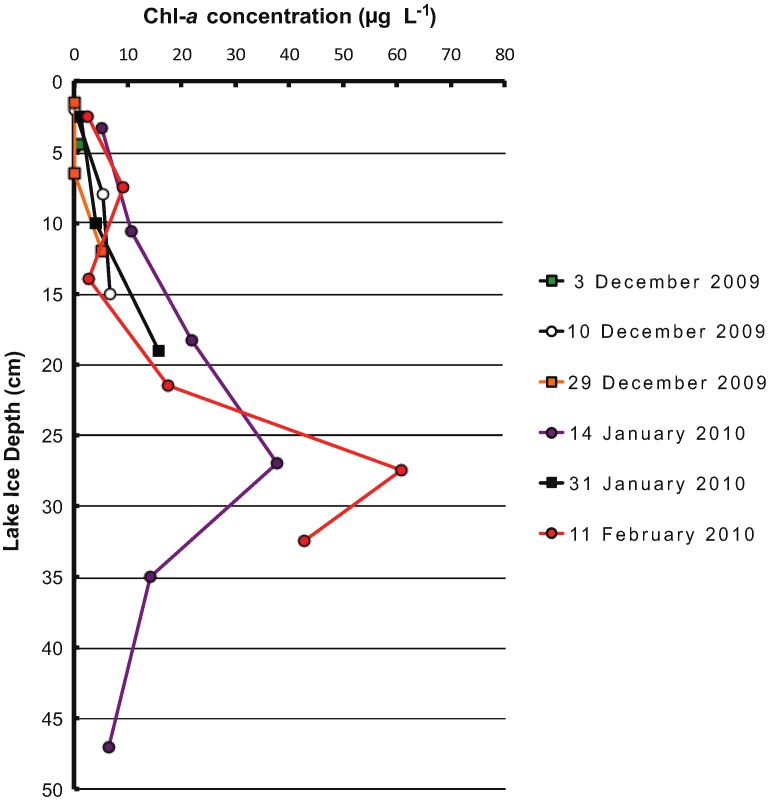
Chl-*a* distribution in Miquelon Lake ice on six sampling dates throughout the 2009–2010 winter season: 3 December 2009, 10 December 2009, 29 December 2009, 14 January 2010, 31 January 2010, and 11 February 2010. Depths indicated are the midpoint of ice core segment processed for biological sampling.

There was a sustained Chl-*a* peak at a depth of ~0.25 to 0.3 m throughout the season (Figure 2). The peak, which was 2 to 2.5 times higher at this depth than at any other depth in the core, was sustained for the months where ice was thick enough to reach this depth despite flushing of the brine and ice growth.

**Figure 3:**
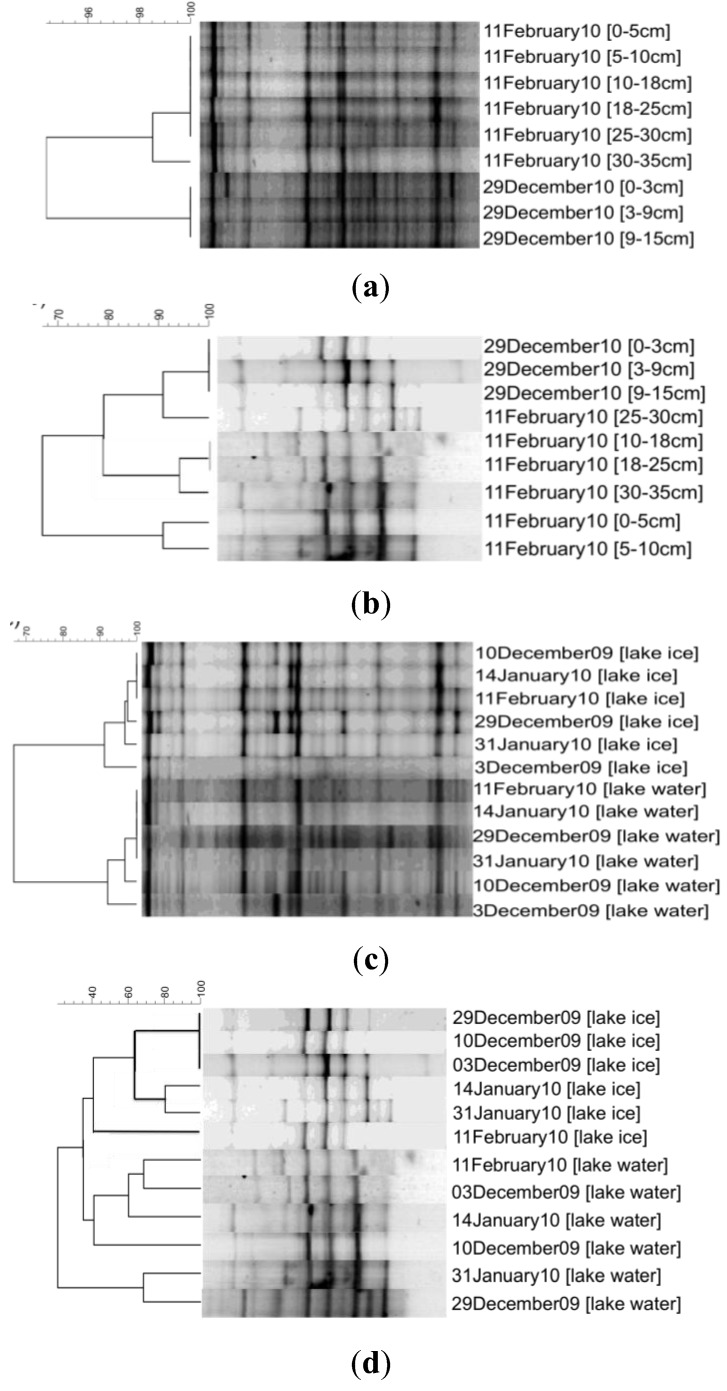
Cluster analysis of DGGE profiles from Miquelon Lake. Cladogram generated by Unweighted Pair Group Method with Arithmetic Mean (UPGMA) of Dice correlation coefficients (which reflect only band presence/absence, not band intensity). (**a**) Similarity of depth profile of Bacteria 16S rRNA genes in lake ice from two representative dates: 29 December 2009 and 11 February 2010. (**b**) Similarity of depth profile of Eukarya 18S rRNA genes in lake ice from two representative dates: 29 December 2009 and 11 February 2010. (**c**) Similarity of Bacteria 16S rRNA genes from homogenized ice cores or water samples from six representative dates: 3 December 2009, 10 December 2009, 29 December 2009, 14 January 2010, 31 January 2010, and 11 February 2010. (**d**) Similarity of Eukarya 18S rRNA genes from homogenized ice cores or water samples from six representative dates: 3 December 2009, 10 December 2009, 29 December 2009, 14 January 2010, 31 January 2010, and 11 February 2010.

DGGE cluster analysis of Bacteria and Eukarya SSU rRNA genes shows a conserved microbial assemblage within the ice (Figure 3). Bacterial assemblages were nearly invariant with depth, both early in the season with thin ice (29 December 2009) and late in the season with thicker ice (11 February 2010) (Figure 3a). The bacterial assemblage was highly similar between the two dates as well, sharing >94% similarity. Ice bacterial assemblages were ~66% similar to those in the underlying water and both were nearly invariant throughout the season (Figure 3c).

Although still highly similar, eukaryotic assemblages were more variable with depth, showing >70% similarity; however, this decreased similarity may be an artifact of the small numbers of bands (*i.e.*, changes in a single band could have an outsized impact on similarity) (Figure 3b). Both ice and water eukaryotic assemblages were essentially invariant throughout the season, showing >90% similarity between dates (Figure 3d). The ice eukaryotic assemblages were highly similar to those in the underlying water, showing >80% similarity (Figure 3d). Variability with depth on a single date exceeded that between dates for the eukaryotes, indicating there was minimal change in the eukaryotic assemblage over time (Figure 3b,d). Overall, the eukaryotic assemblage had lower band richness than the bacterial assemblage.

**Figure 4:**
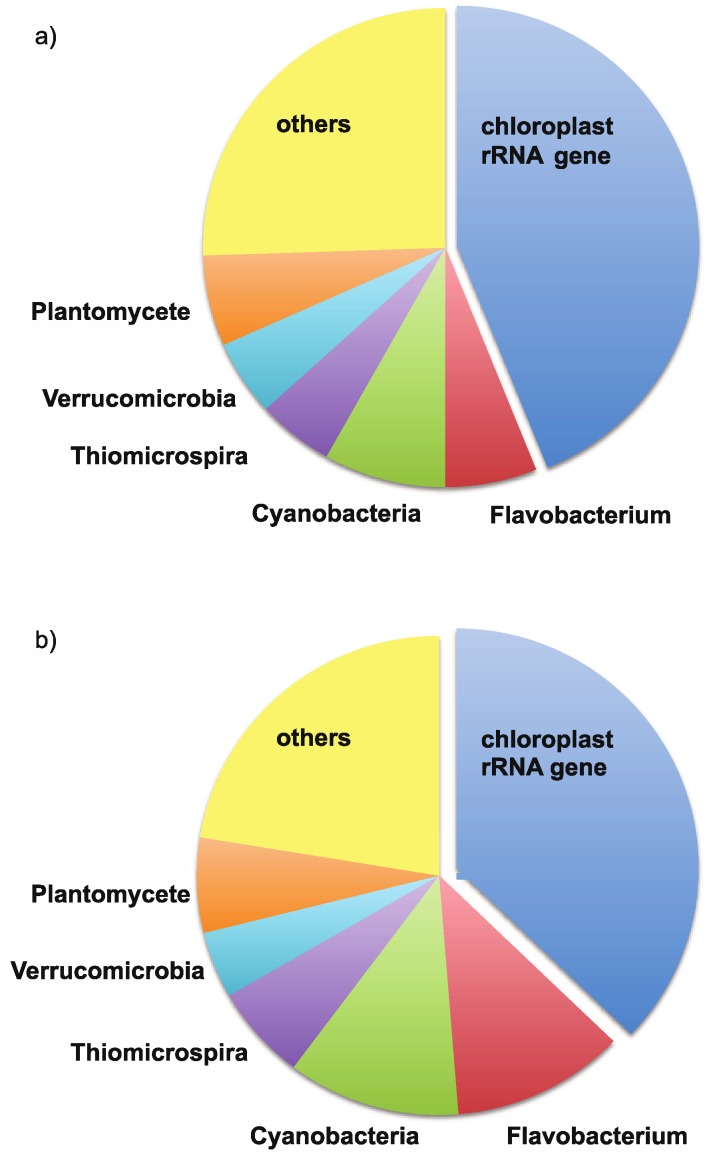
Phylum-level distribution of: (**a**) bacteria ice; and (**b**) bacteria water clone libraries. The “other” category includes all phyla that were represented by <10 clones in each library (see main text for more details). Chloroplast rRNA gene sequences are separated because they were not included in subsequent analyses.

**Figure 5:**
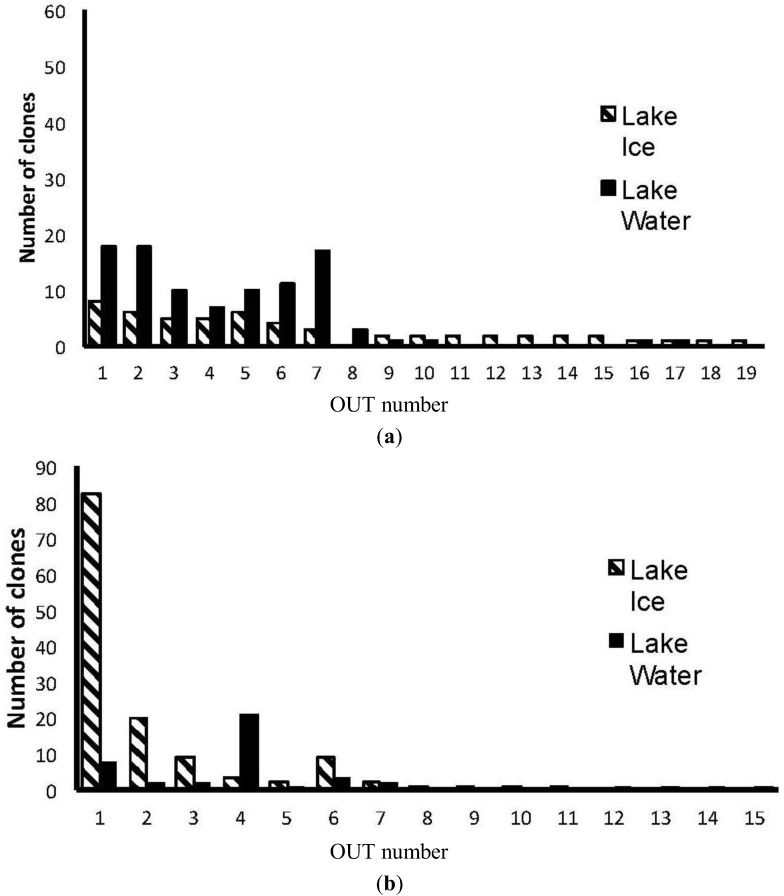
Rank-abundance curves for: (**a**) bacteria; and (**b**) Eukarya clone libraries. Operational taxonomic units (out) were numbered in order of total number of clones in both libraries. Hatched bars show the number of clones in the ice clone library; solid bars show the number of clones in the water clone library.

In order to identify the origin of the dominant bands and elucidate the differences between the ice and water consortia, we constructed a bacterial and eukaryal clone library for the bulked ice (homogenized all ice depths and all sample dates) and bulked water (homogenized all sample dates). The dominant band in the bacterial DGGE (Figure 3a) (initially making up 71% of the bacterial ice clone library and 41% of the bacterial water clone library) was identified as a chloroplast rRNA gene sequence closely related to those from *Nannochloropsis oceanica* and *Chlorella minutissima* (Figure 4). These clones were excluded from further analysis. The Bacteria clone library had a total of 314 clones (with 213 clones remaining after removal of the chloroplast rRNA gene sequences). These clones were grouped into 19 unique operational taxonomic units (OTU) by RFLP analysis. Eleven of these OTU were found in both ice and the underlying waters, with the remainder only present in one of the clone libraries (Figure 5). Miquelon Lake ice and water show a surprising rank abundance curve (Figure 5), with half of the OTUs being represented by roughly equivalent numbers of clones and the remainder of the OTUs comprising a short tail of singletons. A more standard rank-abundance curve, where a few OTUs dominate the clone library and the remaining OTUs are a long tail of singletons, is the pattern seen in the Eukarya rank abundance curve [Eukaryal Lake Ice (n = 123), and Eukaryal Lake Water (n = 39) after removal of the chloroplast rRNA gene sequences] (Figure 5).

**Figure 6:**
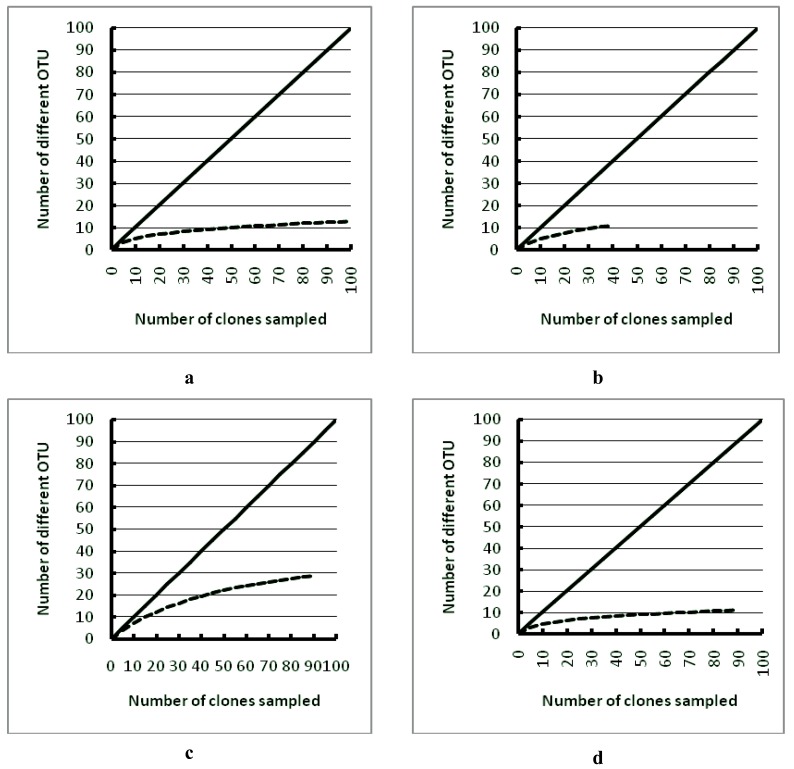
Collector’s curves for: (**a**) Eukarya ice; (**b**) Eukarya water; (**c**) bacteria ice; and (**d**) bacteria water clone libraries. The top line in each graph shows the hypothetical line if each clone belonged to a novel OTU.

Based on rarefaction curves, both the ice and water bacterial diversity was sampled to near completion (Figure 6). Separately, these libraries accounted for 50% and 72% of the overall predicted diversity, based on Good’s coverage estimate for the ice cover and water, respectively; however, the combined estimate for bacterial coverage is 92%. The ice Eukarya diversity was sampled to completion, but the coverage of the water clone library was lower (Figure 6). Good’s coverage estimation agreed with lower coverage for the Eukaryal water library (ice: 95% and water: 37%), but the combined estimate for ice and water had 97.8% coverage.

**Figure 7:**
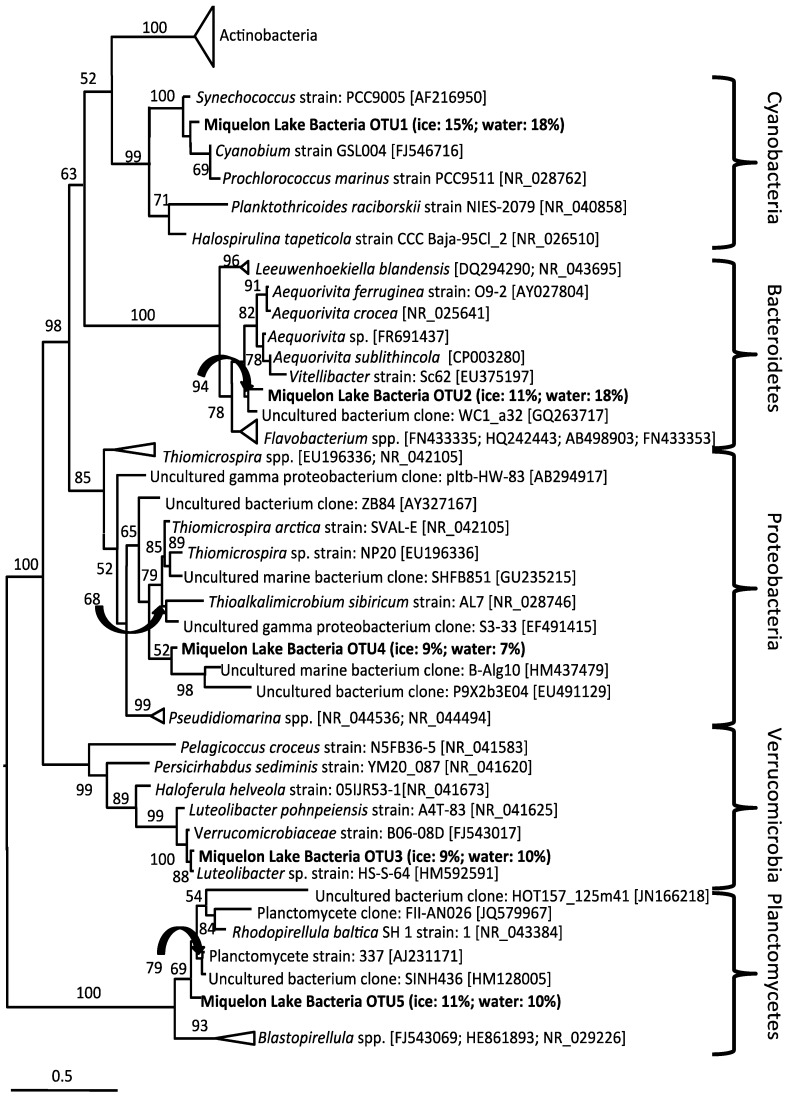
Maximum likelihood phylogenetic tree of Bacteria 16S rRNA genes from Miquelon Lake water and ice and relatives from the Genbank database. Scale bar represents 1 nucleotide change for each 10 nucleotides of sequence. Bootstrap support greater than 50 (of 100 replicates) is shown at nodes. Accession numbers for publically available sequences are given in parentheses. Miquelon Lake OTU are shown in bold; the relative abundance in the ice and water clone libraries is shown in brackets.

**Figure 8:**
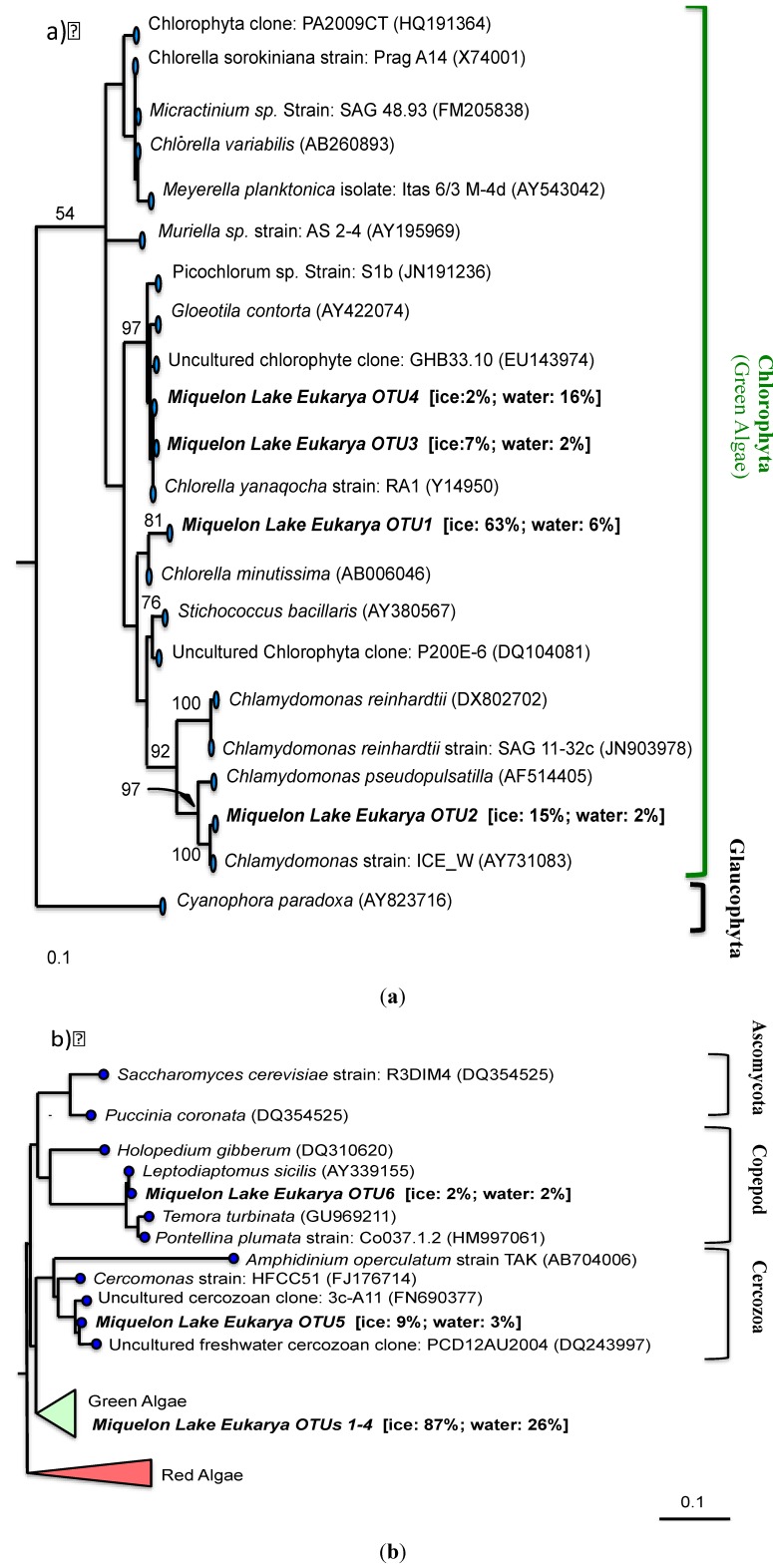
Maximum likelihood phylogenetic tree of Eukarya 18S rRNA genes from Miquelon Lake water and ice and relatives from the Genbank database. Scale bar represents 1 nucleotide change for each 10 nucleotides of sequence. Bootstrap support greater than 50 (of 100 replicates) is shown at nodes. Accession numbers for publically available sequences are given in parentheses. Miquelon Lake OTU are shown in bold; the relative abundance in the ice and water clone libraries is shown in brackets. (**a**) Detail showing relationships of Miquelon Eukarya OTU 1-4 within the Chlorophyta (green algae). (**b**) Phylogenetic position of Miquelon Eukarya OTU 5 and 6 within the Eukarya as a whole.

The closest relatives of the OTUs obtained in the bacterial clone library include the phyla Actinobacteria, Bacteroidetes, Proteobacteria, Verrucomicrobia, and Cyanobacteria (Figure 7). The eukaryotic clone library was dominated almost entirely by OTU with nearest neighbors from green algae, including *Chlamydomonas*, *Chlorella*, and other chlorophytes (Figure 8a). However, a copepod and cercozoan were also identified in both the ice and underlying waters (Figure 8b), indicating the possible presence of a complete microbial food web within the ice-cover of this seasonally frozen lake.

## 4. Discussion

Our findings support four main conclusions: (1) the ice assemblage composition is essentially invariant with depth in the ice; (2) the ice assemblage composition is essentially invariant throughout the season; (3) the ice and lake assemblages might be active throughout the winter; and (4) there may be a complete microbial loop within the ice-cover of this seasonally frozen briny lake in central Alberta.

The invariance in ice assemblage composition with depth might be an indication that the observed variations in temperature, brine volume, and brine salinity (Figure 1) did not have a significant impact on the composition of the microbial ice assemblages. Although Miquelon Lake ice does not reach the extremes sometimes seen in sea ice [1], it is surprising that the variation between brackish (~10 ppt) to hypersaline (>60 ppt) and subzero temperatures did not lead to more obvious changes in the assemblage composition. This lack of variation may be due to a high degree of microbial mixing within the brine channels; a premise supported by the observation that the majority (83%) of the bacterial clones were representatives of OTU that were present in both the lake ice and waters. Mixing events that force underlying water to flush through the brine channels and onto the top of the overlying ice have been observed at Miquelon Lake. The temporal stability of the lake ice and water microbial assemblages demonstrate that time in ice does not affect the microbial composition.

The presence of a seasonally stable Chl-*a* peak indicates some biological activity and growth because this Chl-*a* peak remains at the same ice depth regardless of brine movement and flushing and ice growth and loss, indicating that they must grow in order to maintain the same position. Biological activity may therefore be sustained within the ice throughout the winter, similar to activity that has been observed in sea ice and perennial lake ice [8,24,39]. Over-winter microbial biological activity could theoretically have significant, previously unrecognized implications for nutrient mass balance and productivity estimates in saline lake systems.

It was somewhat surprising that Miquelon Lake clones were not similar to clones and isolates from sea ice or the ice covered lakes of Antarctica. Miquelon Lake ice bacteria were predominantly related to sequences originally found in cold-water lakes, springs, and saline environments. This finding implies that the freezing process does not strongly influence the microbes in the perennial lake ice. Two clones in the lake ice and waters were over 97% similar to human skin biota and wastewater treatment waters, likely representing human contamination. Some contamination might be expected as the source waters for the lake incorporate significant agricultural drainage.

We were unable to consistently detect Archaea in Miquelon lake ice or water by PCR and qPCR measurements indicated that they comprise <1% of the total assemblage. Archaea, which are active in seawater during winter [40], have not been identified consistently within sea ice and represented very low percentages of the total ice-population when found [39]. It is not clear why Archaea appear to be rare in icy environments.

## 5. Conclusions

Briny lakes play important roles in global ecosystem processes. It has been assumed previously that these lakes become essentially biologically inactive in winter and that their ice is biologically inert. Here we have demonstrated that microbes are entrained in the seasonal ice and may maintain biological activity throughout the winter. SSU rRNA genes for primary producers, bacteria, bacteriovores, and bacteriovore predators were observed in Miquelon Lake ice and the underlying water, raising the possibility of a complete microbial loop within the lake ice. These organisms may be actively cycling organic carbon and nutrients throughout the winter. Further studies will help clarify the role of winter microbial ecosystem dynamics in overall ecosystem function and structure.

## References

[1] Mock T., Thomas D.N. (2005). Recent advances in sea-ice microbiology. Environ. Microbiol..

[2] Dieser M., Nocker A., Priscu J.C., Foreman C.M. (2010). Viable microbes in ice: Application of molecular assays to McMurdo Dry Valley lake ice communities. Antarct. Sci..

[3] Foreman C.M., Dieser M., Greenwood M., Cory R.M., Laybourn-Parry J., Lisle J.T., Jaros C., Miller P.L., Chin Y.P., Mcknight D.M. (2011). When a habitat freezes solid: Microorganisms over-winter within the ice column of a coastal Antarctic lake. FEMS Microbiol. Ecol..

[4] Priscu J.C., Fritsen C.H., Adams E.E., Giovannoni S.J., Paerl H.W., McKay C.P., Doran P.T., Gordon D.A., Lanoil B.D., Pinckney J.L. (1998). Perennial antarctic lake ice: An oasis for life in a polar desert. Science.

[5] Bowers J.A., Cooper W.E., Hall D.J. (1990). Midwater and epibenthic behaviors of Mysis relicta Loven: Observations from the Johnson-Sea-Link II submersible in Lake Superior and from a remotely operated vehicle in northern Lake Michigan. J. Plankton Res..

[6] Vanderploeg H.A., Bolsenga S.J., Fahnenstiel G.L., Liebig J.R., Gardner W.S. (1992). Plankton ecology in an ice-covered bay of Lake Michigan: Utilization of a winter phytoplankton bloom by reproducing copepods. Hydrobiologia.

[7] Lizotte M.P. (2001). The contributions of sea ice algae to Antarctic marine primary production. Am. Zool..

[8] Kottmeier S.T., Sullivan C.W. (1988). Sea ice microbial communities (SIMCO)—9. Effects of temperature and salinity on rates of metabolism and growth of autotrophs and heterotrophs. Polar Biol..

[9] McGrath Grossi S., Kottmeier S.T., Sullivan C.W. (1984). Sea ice microbial communities. III. Seasonal abundance of microalgae and associated bacteria, McMurdo Sound, Antarctica. Microb. Ecol..

[10] Garrison D.L., Buck K.R., Silver M.W. (1984). Microheterotrophs in the ice-edge zone. Antarct. J. US.

[11] Kottmeier S.T., Sullivan C.W. (1990). Bacterial biomass and production in pack ice of Antarctic marginal ice edge zones. Deep Sea Res. Oceanogr. Res. Pap..

[12] Last W.M. (2002). Geolimnology of salt lakes. Geosci. J..

[13] Bowman J.S., Sachs J.P. (2008). Chemical and physical properties of some saline lakes in Alberta and Saskatchewan. Saline Syst..

[14] Swanson H., Zurawell R. (2006). Miquelon Lake water quality monitoring report. Provincial Park Lakes Monitoring Program. http://environment.gov.ab.ca/info/library/7730.pdf.

[15] Bierhuizen J.F.H., Prepas E.E. (1985). Relationships between nutrients, dominant ions, and phytoplankton standing crop in prairie saline lakes. Can. J. Fish. Aquat. Sci..

[16] Evans J.C., Prepas E.E. (1996). Potential effects of climate change on ion chemistry and phytoplankton communities in prairie saline lakes. Limnol. Oceanogr..

[17] Hammer U.T. (1978). The Saline Lakes of Saskatchewan I. Background and Rationale for Saline Lakes Research. Int. Rev. Gesamten Hydrobiol. Hydrog..

[18] Haynes R.C., Hammer U.T. (1978). The saline lakes of Saskatchewan. IV Primary production of phytoplankton in selected saline ecosystems. Int. Rev. Gesamten Hydrobiol..

[19] Grasby S.E., Londry K.L. (2007). Supporting a Mid-Continent Marine Ecosystem: An Analogue for Martian Springs?. Astrobiology.

[20] Sørensen K.B., Teske A. (2006). Stratified communities of active archaea in deep marine subsurface sediments. Appl. Environ. Microbiol..

[21] Sorokin D.Y., Tourova T.P., Lysenko A.M., Muyzer G. (2006). Diversity of culturable halophilic sulfur-oxidizing bacteria in hypersaline habitats. Microbiology.

[22] Richter-Menge J.A., Perovich D.K., Elder B.C., Claffey K., Rigor I., Ortmeyer M. (2006). Ice mass-balance buoys: A tool for measuring and attributing changes in the thickness of the Arctic sea-ice cover. Ann. Glaciol..

[23] Cox G.F.N., Weeks W.F. (1983). Equations for determining the gas and brine volumes in sea ice samples. J. Glaciol..

[24] Porter K.G., Feig Y.S. (1980). The use of DAPI for identifying and counting aquatic microflora. Limnol. Oceanogr..

[25] Bergmann M., Peters R.H. (1980). A simple reflectance method for the measurement of particulate pigment in lake water and its application to phosphorus-chlorophyll-seston relationships. Can. J. Fish. Aquat. Sci..

[26] Kulp T.R., Han S., Saltikov C.W., Lanoil B.D., Zargar K., Oremland R.S. (2008). Effects of imposed salinity gradients on dissimilatory arsenate reduction, sulfate reduction, and other microbial processes in sediments from California soda lakes. Appl. Environ. Microbiol..

[27] Myers R., Fischer S.G., Lerman L.S., Maniatis T. (1985). Nearly all single base substitutions in DNA fragments joined to a GC-clamp can be detected by denaturing gradient gel electrophoresis. Nucleic Acids Res..

[28] Díez B., Pedrós-Alió C., Marsh T.L., Massana R. (2001). Application of denaturing gradient gel electrophoresis (DGGE) to study the diversity of marine picoeukaryotic assemblages and comparison of DGGE with other molecular techniques. Appl. Environ. Microbiol..

[29] Bano N., Ruffin S., Ransom B., Hollibaugh J.T. (2004). Phylogenetic Composition of Arctic Ocean Archaeal Assemblages and Comparison with Antarctic Assemblages. Appl. Environ. Microbiol..

[30] Labrenz M., Sintes E., Toetzke F., Zumsteg A., Herndl G.J., Seidler M., Jürgens K. (2010). Relevance of a crenarchaeotal subcluster related to Candidatus Nitrosopumilus maritimus to ammonia oxidation in the suboxic zone of the central Baltic Sea. ISME J..

[31] Polz M.F., Cavanaugh C.M. (1998). Bias in template-to-product ratios in multitemplate PCR. Appl. Environ. Microbiol..

[32] Muyzer G., de Waal E.C., Uitterlinden A.G. (1993). Profiling of complex microbial populations by denaturing gradient gel electrophoresis analysis of polymerase chain reaction-amplified genes coding for 16S rRNA. Appl. Environ. Microbiol..

[33] Delong E.F. (1992). Archaea in coastal marine environments. PNAS.

[34] Skidmore M., Anderson S.P., Sharp M., Foght J., Lanoil B.D. (2005). Comparison of microbial community compositions of two subglacial environments reveals a possible role for microbes in chemical weathering processes. Appl. Environ. Microbiol..

[35] Good I.J. (1953). The population frequencies of species and the estimation of population parameters. Biometrika.

[36] Lanoil B.D., Sassen R., La Duc M.T., Sweet S.T., Nealson K.H. (2001). Bacteria and Archaea Physically Associated with Gulf of Mexico Gas Hydrates. Appl. Environ. Microbiol..

[37] Decipher. http://decipher.cee.wisc.edu/.

[38] Drummond A., Ashton B., Buxton S., Cheung M., Cooper A., Duran C., Field M., Heled J., Kearse M., Markowitz S. Geneious Pro: Geneious v5.4.5; Biomatters. http://www.geneious.com/.

[39] Junge K., Eicken H., Deming J.W. (2004). Bacterial Activity at −2 to −20 °C in Arctic Wintertime Sea Ice. Appl. Environ. Microbiol..

[40] Murray A.E., Wu K.Y., Moyer C.L., Karl D.M., Delong E.F. (1999). Evidence for circumpolar distribution of planktonic Archaea in the Southern Ocean. Aquat. Microb. Ecol..

